# Supplementary material for: Experiences with Family Planning amongst Persons with Mental Health Problems: A Nationwide Patient Survey
Source: Int J Environ Res Public Health. 2023 Feb 9;20(4):3070. doi: 10.3390/ijerph20043070 (PMC9966993; doi:10.3390/ijerph20043070)
Supplement: Supplementary file 1 [file ijerph-20-03070-s001.zip › ijerph-2106724-supplementary.pdf]

Experiences with Family Planning amongst Persons with Mental Health Problems: A Nationwide Patient Survey

Noralie N. Schonewille [1,2,3,*], Monique J. M. van den Eijnden [4], Nini H. Jonkman [5], Anne A. M. W. van Kempen [6], Maria G. van Pampus [7], Francisca G. Goedhart [4], Odile A. van den Heuvel [2,8,9] and Birit F. P. Broekman [1,2,3]

1 Department of Psychiatry and Medical Psychology, OLVG, Oosterpark 9, 1091 AC Amsterdam, The Netherlands
2 Department of Psychiatry, Amsterdam UMC, Location Vrije Universiteit Amsterdam, Boelelaan 1117, 1081 HV Amsterdam, The Netherlands
3 Amsterdam Public Health, Mental Health program, Amsterdam UMC, Location Vrije Universiteit Amsterdam, Boelelaan 1117, 1081 HV Amsterdam, The Netherlands
4 Team Knowledge, Innovation and Research, MIND, Stationsplein 125, 3818 LE Amersfoort, The Netherlands
5 Department of Research and Epidemiology, OLVG, Oosterpark 9, 1091 AC Amsterdam, The Netherlands
6 Department of Neonatology and Pediatrics, OLVG, Oosterpark 9, 1091 AC Amsterdam, The Netherlands
7 Department of Gynecology and Obstetrics, OLVG, Oosterpark 9, 1091 AC Amsterdam, The Netherlands
8 Department of Anatomy & Neuroscience, Amsterdam UMC, Location Vrije Universiteit Amsterdam, Boelelaan 1117, 1081 HV Amsterdam, The Netherlands
9 Amsterdam Neuroscience, Compulsivity, Impulsivity & Attention Program, 1081 HV Amsterdam, The Netherlands
* Correspondence: n.n.schonewille@olvg.nl; Tel.: +31-640978680

Supplementary Table S1 Data supporting Figure 1

Question in survey	Stratified by group	Response category	All respondents N=378 (Former) patients N=354 N	%	P-value*
(1) I have been pregnant in my life or expected a baby (as a partner)	Women (n=295)	Yes	141	47.8	<0.001
		No	154	52.2	
	Men (n=51)	Yes	6	11.8	
		No	45	88.2	
(2) I have children	Women (n=295)	Yes	137	46.4	<0.001
		No	158	53.6	
	Men (n=51)	Yes	6	11.8	
		No	45	88.2	
(3) I have experienced an unintended pregnancy in my life as women or as partner	Women (n=295)	Yes	64	21.7	0.105**
		No	221	74.9	
		Not applicable	4	1.4	
		Missing	*6*	*2.0*	
	Men (n=51)	Yes	4	7.8	
		No	41	80.4	
		Not applicable	1	2.0	
		Missing	5	9.8	
(4) I have had an elective abortion in my life	Women (n=295)	Yes	16	5.4	
		No	279	94.6	

*P-value from Chi^2 test comparing 'yes' category between women and men

** Chi^2 test should be interpreted with caution as numbers in cell are less than 5

Supplementary Table S2 Data supporting Figure 2

		All respondents N=378				Respondents with children N=151				Respondents without children N=227				
		(Former) patients N=354		Close ones N=24		(Former) patients N=147		Close ones N=4		(Former) patients N=207		Close ones N=20		
		N	%	N	%	N	%	N	%	N	%	N	%	P-value*
(1) Mental problems influenced my desire to have children	Yes	196	55.4	11	45.8	40	27.2	0	0.0	156	75.4	11	55.0	< 0.001
	No	104	29.4	6	25.0	73	49.7	3	75.0	31	14.8	3	15.0	
	I am not sure	24	6.8	2	8.3	12	8.2	0	0.0	12	5.8	2	10.0	
	Other	27	7.6	5	20.8	21	14.3	1	25.0	6	2.9	4	20.0	
	Missing	*3*	*0.0*	*0*	*0.0*	*1*	*0.7*	*0*	*0.0*	*2*	*1.0*	*0*	*0.0*	
(2) Mental problems influenced my desire to extend my family after the first child (persons with children)	Yes					46	31.3	1	25.0					
	No					56	38.1	2	50.0					
	I am not sure					10	6.8	1	25.0					
	Maybe					14	9.5	0	0.0					
	Not applicable					17	11.6	0	0.0					
	Missing					*4*	*2.7*	*0*	*0.0*					
(3) Mental problems influenced my ability to become pregnant	Yes	101	28.5	7	29.2	25	17.0	0	0.0	76	36.7	7	35.0	<0.001
	No	183	51.7	6	25.0	98	66.7	3	75.0	85	41.1	3	15.0	
	I am not sure	28	7.9	5	20.8	8	5.4	1	25.0	20	9.7	4	20.0	
	Other	35	9.9	5	20.8	11	7.5	0	0.0	24	11.6	5	25.0	
	Missing	*7*	*2.0*	*1*	*4.2*	*5*	*3.4*	*0*	*0.0*	*2*	*1.0*	*1*	*5.0*	
(4) My childlessness is related to my mental problems	Yes									100	48.3	9	45.0	
	No									17	8.2	4	20.0	
	I am not sure									20	9.7	0	0.0	
	Other									31	15.0	0	0.0	
	Not applicable									0	0.0	1	5.0	
	Missing									*39*	*18.9*	*6*	*30.0*	
(5) Mental problems influenced my family planning	Yes	91	25.7	4	16.7	40	27.2	1	25.0	51	24.6	3	15.0	0.673
	No	129	36.5	4	16.7	75	51.0	2	50.0	54	26.1	2	10.0	
	I am not sure	26	7.3	1	4.2	13	8.8	0	0.0	13	6.3	1	5.0	
	Not applicable	102	28.8	14	58.3	15	10.2	1	25.0	87	42.0	13	65.0	
	Missing	*6*	*1.7*	*1*	*4.2*	*4*	*2.7*	*0*	*0.0*	*2*	*1.0*	*1*	*5.0*	
(6) I had a conversation about family planning when desired	Yes	77	21.8	4	16.7	20	13.6	0	0.0	57	27.5	4	20.0	0.003
	No	140	39.5	6	25.0	45	30.6	1	25.0	95	45.9	5	25.0	
	I am not sure	9	2.5	5	20.8	3	2.0	0	0.0	6	2.9	5	25.0	
	Other	18	5.1	2	8.3	6	4.1	0	0.0	12	5.8	2	10.0	
	Not applicable	88	24.9	5	20.8	58	39.5	1	25.0	30	14.5	4	20.0	
	Missing	*22*	*6.2*	*2*	*8.3*	*15*	*10.2*	*2*	*50.0*	*7*	*3.4*	*0*	*0.0*	
(7) I had a conversation about family planning when desired		N=107 (Former) patients		N=6 Close ones										
Age <40 years	Yes	41	38.8	1	16.7									
	No	49	45.8	2	33.3									
	I am not sure	2	1.9	2	33.3									
	Other	5	4.7	0	0.0									

	Not applicable	8	7.5	1	16.7									
	Missing	*2*	*1.9*	*0*	*0.0*									
		N=222 (Former) patients		N=13 Close ones										
Age >40 years	Yes	30	13.5	3	23.1									
	No	78	35.1	3	23.1									
	I am not sure	7	3.2	2	15.4									
	Other	12	5.4	1	7.7									
	Not applicable	76	34.2	3	23.1									
	Missing	*19*	*8.6*	*1*	*7.7*									
(8) I experienced a taboo around discussing my mental health problems	Yes	221	62.4	6	25.0	94	63.9	2	50.0	127	61.4	4	20.0	0.700
	No	94	26.6	15	62.5	41	27.9	1	25.0	53	25.6	14	70.0	
	I am not sure	23	6.5	2	8.3	8	5.4	1	25.0	15	7.2	1	5.0	
	Other	23	3.7	1	4.2	3	2.0	0	0.0	10	4.8	1	5.0	
	Missing	*3*	*0.8*	*0*	*0.0*	*1*	*0.7*	*0*	*0.0*	*2*	*1.0*	*0*	*0.0*	

*P-value from Chi^2 test comparing 'yes' category between (former) patients with children ('yes') and (former) patients without children

Supplementary Table S3 Data supporting Figure 3

		All respondents N=378				Respondents with children N=151				Respondents without children N=227			
		(Former) patients n=354		Close ones n=24		(Former) patients N=147		Close ones N=4		(Former) patients N=207		Close ones N=20	
		N	%	N	%	N	%	N	%	N	%	N	%
(1) My mental health situation has been related to my pregnancies or first years of parenthood	Yes					75	51.0	0	0.0				
	No					51	34.7	4	100				
	I am not sure					11	7.5	0	0.0				
	Not applicable					7	4.8	0	0.0				
	Missing					*3*	*2.0*	*0*	*0.0*				
(2) My mental health has impacted the live(s) of my child(ren)	Yes					49	33.3	1	25.0				
	No					37	25.2	1	25.0				
	I am not sure					17	11.6	0	0.0				
	Maybe					34	23.1	2	50.0				
	Not applicable					7	4.8	0	0.0				
	Missing					*3*	*2.0*	*0*	*0.0*				
(3) I felt supported when my mental health impacted the live(s) of my child(ren)*	Yes					24	49.0	1	100.0				
	No					25	51.0	0	0.0				
	Missing					*0*	*0.0*	*0*	*0.0*				
(4) There were times when, during my pregnancy or after my delivery, I thought my mental health problems impaired me as a parent	Yes					88	59.9	1	25.0				
	No					37	25.2	1	25.0				
	I am not sure					11	7.5	1	25.0				
	Not applicable					10	6.8	0	0.0				
	Missing					*1*	*0.7*	*1*	*25.0*				
(5) My partner supported me in my parenthood	Yes					69	46.9	3	75.0				
	No					42	28.6	1	25.0				
	I am not sure					14	9.5	0	0.0				
	Not applicable					21	14.3	0	0.0				
	Missing					*1*	*0.7*	*0*	*0.0*				

* Calculated amongst 49 persons who answered yes on question (2) Figure 3

Supplementary Table S4 Data supporting Figure 4

		All respondents N=378				Respondents with children N=151				Respondents without children N=227				
		(Former) patients n=354		Close ones n=24		(Former) patients N=147		Close ones N=4		(Former) patients N=207		Close ones N=20		
		N	%	N	%	N	%	N	%	N	%	N	%	P-value*
(1) I experienced a taboo around discussing sexuality	Yes	206	58.2	5	20.8	82	55.8	2	50.0	124	59.9	3	15.0	0.506
	No	137	38.7	19	79.2	60	40.8	2	50.0	77	37.2	17	85.0	
	Missing	*11*	*3.1*	*0*	*0.0*	*5*	*3.4*	*0*	*0.0*	*6*	*2.9*	*0*	*0.0*	
(2) There was a period in which I had mental problems, was sexually active and had no intention to become pregnant	Yes	223	63.0	12	50.0									
	No	54	15.3	0	0.0									
	I am not sure	22	6.2	5	20.8									
	Not applicable	47	13.3	7	29.2									
	Missing	*8*	*2.3*	*0*	*0.0*									
(3) I always used contraceptives when (my partner was) at risk or pregnancy**	Yes	168	75.3	10	83.3									
	No	45	20.2	1	8.3									
	I am not sure	8	3.6	1	8.3									
	Missing	*2*	*0.9*	*0*	*0.0*									
(4) Mental health problems have influenced my sexual enjoyment	Not at all	29	8.2	4	16.7	14	9.5	0	0.0	15	7.2	4	20.0	
	A little	52	14.7	1	4.2	20	13.6	1	25.0	32	15.5	0	0.0	
	Sometimes	47	13.3	6	25.0	19	12.9	0	0.0	28	13.5	6	30.0	
	Often	92	26.0	6	25.0	38	25.9	0	0.0	54	26.1	6	30.0	
	Very often	122	34.5	6	25.0	50	34.0	2	50.0	72	34.8	4	20.0	0.971
	Missing	*12*	*3.4*	*1*	*4.2*	*6*	*4.1*	*1*	*25.0*	*6*	*2.9*	*0*	*0.0*	

** Calculated amongst 235 persons who answered 'yes' in question (2) Figure 4

*P-value from Chi^2 test comparing 'yes' or 'Very often' category between (former) patients with children and (former) patients without children
